# Accuracy and Reproducibility of Conformal Radiotherapy using Data from a Randomised Controlled Trial of Conformal Radiotherapy in Prostate Cancer (MRC RT01, ISRCTN47772397)

**DOI:** 10.1016/j.clon.2008.04.019

**Published:** 2008-10

**Authors:** S. Stanley, S. Griffiths, M.R. Sydes, A.R. Moore, I. Syndikus, D.P. Dearnaley, RT01 Radiographer Trial Implementation Group on behalf of all the RT01 Collaborators

**Affiliations:** ∗ St James's Institute of Oncology, Leeds, UK; † MRC Clinical Trials Unit, London, UK; ‡ Royal Marsden Hospital, Fulham Road, London, UK; § Clatterbridge Centre for Oncology, Wirral, UK; ∥ Institute for Cancer Research and Royal Marsden Hospital, Sutton, London, UK

**Keywords:** Conformal radiotherapy, controlled trial, portal imaging, prostate cancer, reproducibility

## Abstract

**Aims:**

The MRC RT01 trial used conformal radiotherapy to the prostate, a method that reduces the volume of normal tissue treated by 40–50%. Because of the risk of geographical miss, the trial used portal imaging to examine whether treatment delivery was within the required accuracy.

**Material and methods:**

In total, 843 patients were randomly assigned to receive 64 Gy in 32 fractions over 6.5 weeks or 74 Gy in 37 fractions over 7.5 weeks. Field displacements and corrections were recorded for all imaged fractions. Displacement trends and their association with time, disease and treatment set-up characteristics were examined using univariate and multivariate analyses. A Radiographer Trial Implementation Group (RTIG) was set up to inform the quality assurance process and to promote the development of best practice.

**Results:**

Treatment isocentre positioning was within 5 mm in every direction on 6238 (83%) of the 7535 fractions imaged. In total, 532 (81%) of 695 included patients had at least one ≥ 3mm displacement and 415 (63%) had at least one ≥ 5mm displacement. Univariate, multivariate and stepwise models of ≥ 5mm displacements showed an increased likelihood of displacement in weeks 1 and 2 with low melting point alloy (LMPA) blocks compared with multileaf collimators, film verification compared with electronic portal imaging (EPI) and increased number of fractions imaged. Except for LMPA, this was also seen for ≥ 5mm displacements in weeks 3–6.

**Conclusions:**

Accurate conformal treatment was delivered. The use of EPI was associated with increased reported accuracy. The RTIG was a crucial part of the quality assurance process.

## Introduction

Conformal radiotherapy is now a standard treatment for localised carcinoma of the prostate [1]. The MRC RT01 trial investigated the efficacy and safety of delivering dose-escalated conformal field radiotherapy for prostate cancer [2–4]. Between January 1998 and December 2001, 843 patients at 19 radiotherapy centres (17 in the UK) were randomised to receive either the standard dose (64 Gy/32 fractions) or an escalated dose (74 Gy/37 fractions). This was given as 64 Gy to the prostate and seminal vesicles (phase I) according to risk group, with or without a 10 Gy boost to the prostate only (phase II). Compared with conventional radiotherapy, conformal radiotherapy reduces the volume of normal tissue treated by 40–50% [5]. However, with this comes the risk of geographical miss due to set-up uncertainties. Therefore, regular portal imaging and image analysis was carried out to ensure that treatment delivery was within the margin of tolerance. Displacements were identified and corrections confirmed according to the protocol.

The trial quality assurance group initiated a dosimetric and geometric quality assurance review process [6]. Completion of a quality assurance questionnaire was a prerequisite to participation. The validity of the questionnaire responses was confirmed by visits to all UK centres during the trial where under experimental conditions using a phantom a low and acceptable variation in dosimetric and geometric uncertainty was indicated [7]. The quality assurance processes showed that all centres could deliver and verify conformal radiotherapy to the standard required to participate in the trial.

A Radiographer Trial Implementation Group (RTIG) involving radiographers from all UK participating centres was established to co-ordinate clinical aspects of the trial quality assurance process. RTIG roles included the implementation of radiographer-led portal imaging analysis, ensuring that data on treatment accuracy were collected according to protocol, monitoring technique accuracy in treatment delivery and developing and sharing best practice methods for the radiotherapy process. Displacement and correction data were collected on a radiographers' log case report form for each patient [2,8]. This paper uses the displacement and correction data submitted by the participating centres to assess the variability in the patient set-up and to comment on the accuracy of conformal radiotherapy treatments delivered during only phase I of trial radiotherapy, which was common to all trial patients.

## Materials and Methods

Of 831 patients who started radiotherapy, 824 had radiographer logs returned. However, one participating centre used online imaging to verify and correct before each fraction and so no displacement data were submitted. Therefore, patients from this centre were excluded from these analyses and displacement and correction data for 695 patients were analysed.

### Treatment Technique

The trial protocol allowed for three- or four-field techniques using either multileaf collimators (MLCs) or low melting point alloy (LMPA) shaped blocks (Table 1). All fields were to be treated daily on a linear accelerator of ≥5 MV. All patients were treated supine with locally standard immobilisation.

## Computed Tomography Planning and Safety Margins

Gross tumour volume and clinical target volume were to be defined on computed tomography scans taken at 5 mm intervals from the bottom of the sacro-iliac joints to the penile urethra (1 cm below ischial tuberosities). The clinical target volume was defined as gross tumour volume + 0.5 cm and planning target volume with a three-dimensional safety margin around the clinical target volume of 0.5–1.0 cm. Each participating centre could specify their own planning target volume margin within this range to account for local set-up uncertainties. No oral, rectal or intravenous contrast agents were allowed.

### Verification Protocol, Radiographers' Log Displacement and Correction Data

Although in 1998 electronic portal imaging (EPI) was a recent innovation, used in relatively few UK centres, it is now an established method for determining set-up accuracy [9–13]. For the RT01 trial, an image-based verification protocol was devised to measure set-up displacements and corrections at regular intervals throughout the course of treatment. The radiographers' log used in a previous single-centre pilot study [14] was adapted to record displacement and correction data.

The trial imaging protocol defined a field placement tolerance of 3 mm in any field axis. Positioning errors ≥5 mm were considered unacceptable and were required to have a correction applied before the subsequent fraction being delivered. Images taken, measurements and corrections made and accuracy on the fraction after correction were recorded on the radiographers' log for each fraction imaged. Displacements were recorded for lateral, longitudinal and vertical directions from anterior/posterior and lateral/oblique views. Rotational errors were not recorded as not all centres were capable of accurately quantifying this type of error.

The trial imaging protocol also defined image frequency and megavoltage images were acquired on at least two consecutive fractions during the first week of treatment and once weekly thereafter, with repeat images after any corrections to verify the change. The timing of the image acquisition (before, during or after treatment delivery) was not defined in the protocol. All megavoltage images were compared with either a simulator film or a digitally reconstructed radiograph to determine displacements. The method of image registration used was the choice of the centre. As both film and electronic images were acquired, manual (light box) or software analysis methods were used to measure displacements.

### Statistical Considerations

The direction and size of displacements and corrections were summarised according to the three cardinal axes. Fractions were classified as having a ≥3 mm or ≥5 mm displacement or correction in any one dimension (direction) or over all three dimensions. The number of fractions with and without a displacement, and the number of patients with and without displacement in a given week of treatment were summarised graphically.

It was anticipated that displacements might occur throughout treatment, that set-up errors would most probably occur in the first 2 weeks of treatment, and that corrections would prevent systematically occurring errors thereafter. Univariate and multivariate ordered logistic regression models were run for: patients with at least one ≥3 mm displacement; patients with at least one ≥5 mm displacement; data in weeks 1 and 2; data in weeks 3 onwards. The effects of disease and treatment set-up factors were investigated. The risk group, which was calculated from T-stage, differentiation and prostate-specific antigen, was a stratification factor for the trial randomisation. Therefore, this was included in the multivariate analyses and, consequently, T-stage and differentiation were not included in the multivariate analyses (prostate-specific antigen was not included in either uni- or multivariate analyses). The models considered the impact baseline bladder symptoms, which were dichotomised to none or some according to the Royal Marsden Hospital scale [3]. The treatment centre was not included as a variable in the analyses because the number of centres was too large and there were too many variables within the data from each centre. The allocated dose group was included in the univariate analyses for completeness and assurance, but excluded from the multivariate analyses: these analyses include only data from phase I of treatment, which was identically planned regardless of the allocated trial treatment (treatment differed in terms of the inclusion or not of phase II treatment). Therefore, any effect from the dose group could only be due to chance. There was no evidence of an effect in the univariate analyses for allocated dose group and the factor was not included in the multivariate model. Patients were excluded from the (ordered) logistic regression analyses where imaging was carried out using both EPI and film (*n* = 41) or a six-field technique (*n* = 1) was used. Therefore, data were available for these analyses from 657 of 695 patients.

All analyses were carried out using the statistical package Stata 9 (Stata Corporation, College Station, TX, USA).

## Results

### Translational Displacements

**Fig. 1:**
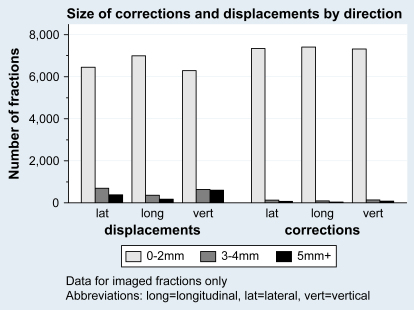
Size and direction of reported corrections and displacements in imaged fractions.

Displacements ≥3 mm were recorded for 2797 (37%) of the 7535 fractions reported. The displacements were similarly distributed across the three directions (Fig. 1) with no good evidence of a difference from zero. The absolute lateral mean displacement was 0.2 mm to the right (standard deviation 2.2 mm); the longitudinal absolute mean displacement was 0.2 mm superiorly (standard deviation 2.4 mm); and the vertical absolute mean displacement was 0.1 mm anteriorly (standard deviation 1.2 mm).

Treatment was delivered within 5 mm of the planned position in every dimension for 6238 (83%) of the 7535 fractions reported, based on the position of bony landmarks. Table 2 shows data by centre. In total, 532 (81%) of 695 patients included had at least one ≥3 mm displacement and 415 (63%) had at least one ≥5 mm displacement.

**Fig. 2:**
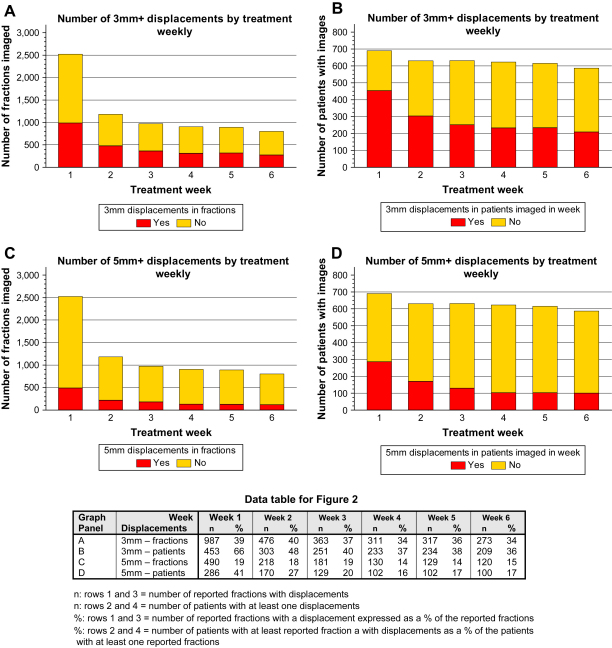
Four-way panel graphs of displacements by week.

The number of fractions imaged reduced over the treatment weeks. This is also reflected in a decrease in the number of patients with fractions imaged. This cannot be explained by patients stopping treatment early. The proportion of reported displacements in a given treatment week is summarised in Fig. 2. The first two weeks had a higher reported proportion of patients with displaced fractions. A similar pattern was seen for the proportion of displacements of ≥3 mm and ≥5 mm. There were fewer corrections than displacements, but the same trend towards decreased corrections carried out by week of treatment was observed (data not shown).

### Association of Disease and Treatment Set-up Characteristics with Displacements

Radiotherapy delivery methods were used in only a small number of combinations across the trial centres and associations were present between the beam modification and treatment verification methods. For example, 407 (96%) patients whose treatment was verified with EPI had beam modification with MLC, whereas this was the case for only 71 (31%) patients verified with film. As expected, the number of images taken was much higher for patients imaged with EPI (mean 27.8, standard deviation 9.0) rather than film (mean 19.6, standard deviation 5.2; *P* < 0.001) and also for MLC (mean 26.8, standard deviation 9.6) over LMPA (mean 21.0, standard deviation 4.4) as the beam modification method (*P* < 0.001).

In the univariate models of ≥3 mm reported displacements in weeks 1 and 2 (Table 3), there was evidence of an increased likelihood of a displacement being reported with higher T-stage, lower age, moderate risk group, beam modification with LMPA, treatment verification with film, four phase I fields and increasing number of imaged fractions reported. In the multivariate model of ≥3 mm reported displacements in weeks 1 and 2, lower age, moderate risk group, treatment verification with film and an increased number of fractions imaged were significant. In weeks 3–6 (Table 4), only an increasing number of fractions reported and treatment verification with film were associated with increased occurrence of at least one reported displacement.

In the univariate, multivariate and stepwise models of ≥5 mm reported displacements there was evidence of an increased likelihood of a displacement across weeks 1–2 and 3–6 with film and an increased number of fractions imaged. LMPA beam modification was associated with increased displacements in all the models except multivariate weeks 3–6 (Tables 5 and 6).

There was no evidence of an effect from the allocated trial treatment, actual dose given or the presence of baseline bladder symptoms.

## Discussion

MRC RT01 was a pragmatic trial that accompanied the initiation of conformal radiotherapy for prostate cancer in many UK centres. The results of external measurements of displacements and associated corrections show that it is possible to give accurate and reproducible treatment. There may be some factors that affect accuracy.

The requirement to correct all errors ≥5 mm at or before the next fraction was applied inconsistently, as centres had differing local correction practices. This will have influenced the number of displacements and corrections reported. Only one centre corrected most of the displacements reported, although the number of displacements reported by this centre was comparatively small. In other centres, corrections were only made after two fractions showing errors of >3 or >5 mm had been measured. This would explain why some centres have more displacements recorded than corrections and is particularly relevant for weeks 2–6, as any systematic errors should have been identified and corrected during week 1 (or at least by week 2). Displacements are more useful here than corrections because displacements were more objective across the trial, whereas corrections required human interpretation and intervention: there were no consistent trial-wide guidelines on how these should be applied. The method of correcting only after errors were seen on two or more fractions is supported by several studies, suggesting that, in order to optimise the correction in terms of identifying the systematic component, repeated imaging is required [15–17]. Assessment of random and systematic errors should be included for future trials.

Local policies for the magnitude of the correction ranged between correcting 50 and 100% of the measured displacements. For this pragmatic trial it was appropriate to allow centres to be comfortable with their own verification policies as many were just starting to use EPI and associated software. To have been more specific may have resulted in poor compliance. However, for any new trial, a more detailed correction policy should be specified. Deviation from the weekly imaging protocol was identified in that the number of patients imaged in a given week was often less than the number of patients still undergoing radiotherapy. The quality assurance visits identified that at least one centre had interpreted the protocol incorrectly and had not imaged weekly during phase 1 for patients treated early in the trial.

The method used to image also had implications. Film verification increased the likelihood of at least one reported displacement compared with EPI, despite the fact that film was associated with significantly fewer displaced fractions reported than EPI and fewer images taken during treatment than EPI. The methods used to measure displacement may also have had an effect. The difficulty in the evaluation of lateral images was highlighted by RTIG. Perera *et al.* [18] found that human observers have difficulty in identifying displacements of <5 mm when using manual methods. It should be noted that where EPI was used in this trial, the software analysis was not based on fully automatic registration.

The displacement data are comparable with those reported in several smaller studies of prostate conformal radiotherapy reproducibility [19–27], where immobilisation methods have been compared. A broad range of displacements was seen between the participating centres and this can be linked to their practice. Further discussion on the methods used by participating centres is discussed in other RT01 publications with recommendations [6,8].

Only one centre was using clinician-led portal image analysis for the trial, although several undertook clinician review after radiographer-led analysis. For most radiographers, this trial provided an opportunity to develop skills in portal imaging and further showed that this is a role that can be successfully undertaken by radiographers [28]. The existence of RTIG provided an unprecedented opportunity for radiographers from a number of centres to discuss treatment delivery, verification and reproducibility issues and to learn from the experiences and practices of others for conformal prostate set-up, enabling developments within centres. It also showed that there is a considerable learning and preparation time and a need for ongoing work to implement new and complex practice, and the need for appropriate technology and associated user skills to be available. It is recommended that for future trials involving technical developments in radiotherapy, a radiographer group is included and this is being undertaken in the UK as part of the Academic Clinical Oncology and Radiobiology Research Network (ACORRN) project [29]. The RTIG forum allowed differences in practice to be explored and best practice was developed [6].

A time trend has been found to be significant in other much smaller studies [23,30]. This was not tested in this trial because of the structure of the available data, but the proportion of imaged fractions associated with a displacement seemed to have decreased over time. However, the clinical significance of any time trend may be questioned. Weekly imaging protocols must balance increased radiation dose and workload with the benefit of accurate treatment associated with the identification and subsequent correction of displacements. It also depends on the tolerance level applied. For a 5 mm tolerance level, the value of weekly imaging is reduced, but for 3 mm tolerance, weekly imaging is recommended.

From the (ordered) logistic regression modelling there was evidence that increased T-stage was associated with increased ≥3 mm displacements reported. We speculate that patients with more symptomatic advanced cancers may have had more difficulty in maintaining a full bladder and therefore increased difficulty in maintaining a stable position during treatment. A more manageable drinking protocol of less than 500 ml or treating with an empty bladder may improve reproducibility, but this would require further study to assess the effects on small bowel and bladder toxicity. This category of patients may be considered unsuitable for further margin reduction techniques unless image guidance is available.

Beam modification with LMPA rather than MLC was associated with increased identification of displacement. The use of LMPA and film may be associated with a longer overall fraction delivery time, so the likelihood of patient movement is increased, combining film and LMPA will compound this effect.

Another consistently important factor was the actual number of fractions reported during each period. There was clear evidence in univariate and multivariate models that an increased number of fractions imaged was statistically significantly associated with the finding of at least one displacement. The number of fractions imaged is not an entirely independent variable; finding displacements or making corrections required further imaging, affecting the total number of fractions imaged and possibly the number of further displacements reported. For these reasons the models focused on the reporting of one reported displacement or more rather than the actual number of displacements. Future trials recording displacement and correction data could pre-specify the fractions for imaging, these then being the primary focus of any analyses of displacement.

## Conclusions

It is feasible to give accurate and reproducible treatment according to external measurements of displacement. The RT01 trial provided a unique opportunity to study the accuracy of treatment techniques being used for conformal radiotherapy on a large scale and showed that patient set-up uncertainties contributed the largest component of the reported errors. Moderate risk group patients were more likely to have a displacement >3 mm. The use of EPI and MLC are associated with fewer displacements reported and weekly imaging is recommended for departments using a 3 mm tolerance level for displacement correction. In future trials where set-up reproducibility is an outcome measure, it would be advantageous to use specified fractions to record displacements and to be more prescriptive in the protocol for the correction of errors to enable random and systematic errors to be assessed.

The RTIG was vital to ensuring that the trial protocol was followed and best practice developed and it is recommended that such a group is included in future similar trials. Radiographer-led assessment of treatment verifications has enabled a UK national multi-centre trial of conformal prostate radiotherapy to be accomplished with acceptably high standards of accuracy and reproducibility.

## Figures and Tables

**Table 1:** Methods used for treatment and verification by centre

Centre*	Number of fields	Beam modification	Image type	Analysis method
2	Four-field	MLC	EPI	Software
3	Both	Both	Both	Software
4	Four-field	MLC	EPI	Software
7	Three-field	MLC	EPI	Software
8	Four-field	MLC	EPI	Software
9	Three-field	MLC	Film	Manual
10	Three-field	MLC	EPI	Software
11	Three-field	Both	EPI	Software
15	Three-field	LMPA	Film	Manual
16	Four-field	MLC	Film	Manual
17	Three-field	MLC	EPI	Software
18	Four-field	LMPA	EPI	Software
19	Three-field	MLC	EPI	Manual
20	Four-field	LMPA	Film	Manual
22	Three-field	LMPA	Film	Manual
23	Three-field	MLC	EPI	Software
25	Three-field	Both	Film	Manual
30	Four-field	MLC	Both	Software

MLC, multileaf collimator; LMPA, low melting point alloy; EPI, electronic portal imaging.

∗ Sites have been coded. The same codes are used in Tables 1 and 2.

**Table 2:** Corrections and displacements overall

Radiotherapy treatment centre*	Patients with data	Total fractions reported	Fractions reported per patient		Total films/images	Displaced fractions reported [*n* (%)]†		Corrected fractions reported [*n* (%)]†	
			Median	Quartiles		3 mm+	5 mm+	3 mm+	5 mm+
2	49	653	13	12–14	1396	246 (38)	38 (6)	11 (2)	3 (0)
3	149	1614	11	9–12	3259	983 (61)	558 (35)	173 (11)	48 (3)
4	35	376	10	10–12	1193	228 (61)	137 (36)	21 (6)	8 (2)
7	22	299	14	12–15	702	102 (34)	41 (14)	34 (11)	7 (2)
8	40	481	11	10–14	968	169 (35)	68 (14)	10 (2)	2 (0)
9	14	137	10	9–11	213	63 (46)	22 (16)	11 (8)	1 (1)
10	40	433	11	10–12	1054	123 (28)	40 (9)	10 (2)	3 (1)
11	139	1518	11	9–13	3204	495 (33)	171 (11)	127 (8)	33 (2)
15	5	29	8	2–8	60	3 (10)	1 (3)	0 (0)	0 (0)
16	31	280	9	8–10	528	85 (30)	49 (18)	35 (13)	19 (7)
17	19	152	8	7–9	364	64 (42)	33 (22)	2 (1)	1 (1)
18	3	23	8	6–9	73	6 (26)	0 (0)	1 (4)	0 (0)
19	92	1028	11	10–12	3213	38 (4)	33 (3)	34 (3)	31 (3)
20	7	61	8	8–10	139	5 (8)	1 (2)	3 (5)	1 (2)
22	22	177	8	7–9	372	9 (5)	2 (1)	1 (1)	1 (1)
23	3	29	10	9–10	87	19 (66)	6 (21)	0 (0)	0 (0)
25	2	23	12	11–12	44	16 (70)	8 (35)	6 (26)	6 (26)
30	23	222	10	8–12	524	143 (64)	89 (40)	11 (5)	11 (5)
Total	695	7535	11	9–12	17 393	2797 (37)	1297 (17)	490 (7)	175 (2)

∗ Sites have been coded. The same codes are used in Tables 1 and 2.

† 3 mm+ displacements include 5 mm+ displacements.

**Table 3:** Patients with one or more displacement of at least 3 mm in weeks 1–2: univariate and multivariate logistic regression models

Category*	Data			Univariate models				Multivariate models				
	Miss	*n*	3 mm+ displacement	OR	(95% CI)	*z*	*P*	*n*	OR	(95% CI)	*z*	*P*
T-stage	2	655	178	1.65	(1.26, 2.17)	3.61	<0.001	645	–	–	–	–
Differentiation	0	657	179	0.81	(0.57, 1.15)	−1.18	0.240	–	–	–	–	–
Age (quartiles)	0	657	179	0.89	(0.76, 1.04)	−1.52	0.129	–	0.84	(0.71, 0.99)	−2.11	0.035
Risk group	0	657	179	1.41	(0.98, 2.02)	1.86	0.063	–	1.49	(1.02, 2.19)	2.06	0.039
Allocated treatment	0	657	179	1.04	(0.74, 1.46)	0.21	0.836	–	1.15	(0.80, 1.64)	0.74	0.461
Dose group	0	657	179	1.00	(0.26, 3.82)	0.00	0.998	–	–	–	–	–
Beam modification	6	651	179	1.41	(0.94, 2.11)	1.65	0.098	–	1.35	(0.74, 2.44)	0.98	0.326
Treatment verification	2	655	179	1.38	(0.96, 2.00)	1.73	0.084	–	2.59	(1.41, 4.75)	3.08	0.002
Phase I fields	2	655	179	1.59	(1.10, 2.30)	2.49	0.013	–	1.40	(0.95, 2.07)	1.72	0.086
Fractions imaged	0	657	179	1.99	(1.50, 2.64)	4.80	<0.001	–	1.47	(1.29, 1.69)	5.63	<0.001
Baseline bladder symptoms	6	651	179	1.05	(0.74, 1.50)	0.28	0.776	–	1.06	(0.73, 1.54)	0.29	0.770

T-stage and differentiation were not included in the multivariate models because they were jointly represented in the risk group. LMPA, low melting point alloy; MLC, multileaf collimator; EPI, electronic portal imaging.

∗ Category (reference category is depicted in bold): T-stage = **T1** vs T2 vs T3 Differentiation = **Good** vs moderate vs poor (based on differentiation or Gleason sum score, where available); Age = 46–62, 63–66, 67–70, 71 and over; Risk group = **Low** vs moderate (trial stratification factor); Allocated treatment = **74Gy/37 fractions** vs 64 Gy/32 fractions; Beam modification = **MLC** vs LMPA; Treatment verification = **EPI** vs film; Phase I fields = **three** vs four (patients with six phase I fields were excluded); Total dose = **<64Gy** vs 64 Gy+; Number of fractions reported = **0–3** vs 4–6 vs 7+.

**Table 4:** Patients with one or more displacement of at least 3mm in weeks 3–6: univariate and multivariate logistic regression models

Category*	Data			Univariate models				Multivariate models				
	Miss	*n*	3 mm+ displacement	OR	(95% CI)	*z*	*P*	*n*	OR	(95% CI)	*z*	*P*
T-stage	2	639	226	1.34	(1.04, 1.72)	2.28	0.023	630	–	–	–	–
Differentiation	0	641	226	0.76	(0.55, 1.07)	−1.58	0.113	–	–	–	–	–
Age (quartile)	0	641	226	1.01	(0.87, 1.17)	0.13	0.900	–	0.96	(0.82, 1.13)	−0.47	0.638
Risk group	0	641	226	1.56	(1.11, 2.19)	2.54	0.011	–	1.32	(0.90, 1.95)	1.43	0.153
Allocated treatment	0	641	226	1.14	(0.83, 1.58)	0.81	0.420	–	1.13	(0.78, 1.62)	0.65	0.518
Dose group	0	641	226	0.45	(0.10, 2.16)	−0.99	0.321	–	–	–	–	–
Beam modification	5	636	224	2.24	(1.50, 3.34)	3.97	<0.001	–	1.00	(0.53, 1.86)	−0.01	0.991
Treatment verification	1	640	226	2.05	(1.43, 2.93)	3.91	<0.001	–	2.01	(1.14, 3.56)	2.4	0.016
Phase I fields	1	640	226	1.92	(1.36, 2.71)	3.70	<0.001	–	1.27	(0.86, 1.88)	1.22	0.222
Fractions imaged	0	641	226	3.98	(2.97, 5.33)	9.28	<0.001	–	1.74	(1.53, 1.98)	8.29	<0.001
Baseline bladder symptoms	6	635	226	0.97	(0.69, 1.37)	−0.15	0.882	–	0.92	(0.63, 1.35)	−0.42	0.677

T-stage and differentiation were not included in the multivariate models because they were jointly represented in the risk group. LMPA, low melting point alloy; MLC, multileaf collimator; EPI, electronic portal imaging.

∗ Category (reference category is depicted in bold): T-stage = **T1** vs T2 vs T3; Differentiation = **Good** vs moderate vs poor (based on differentiation or Gleason sum score, where available); Age = 46–62, 63–66, 67–70, 71 and over; Risk group = **Low** vs moderate (trial stratification factor); Allocated treatment = **74Gy/37 fractions** vs 64 Gy/32 fractions; Beam modification = **MLC** vs LMPA; Treatment verification = **EPI** vs film; Phase I fields = **three** vs four; Total dose = **<64Gy** vs 64 Gy+; Number of fractions reported = **0–3** vs 4–5 vs 6–8 vs 9+.

**Table 5:** Patients with one or more displacement of 5mm in weeks 1–2: univariate and multivariate logistic regression models

Category*	Data			Univariate models				Multivariate models				
	Miss	*n*	3 mm+ displacement	OR	(95% CI)	*z*	*P*	*n*	OR	(95% CI)	*z*	*P*
T-stage	2	655	318	1.07	(0.85, 1.36)	0.59	0.558	645	–	–	–	–
Differentiation	0	657	320	0.81	(0.59, 1.12)	−1.28	0.202	–	–	–	–	–
Age (quartile)	0	657	320	1.02	(0.88, 1.17)	0.23	0.819	–	0.98	(0.84, 1.14)	−0.28	0.778
Risk group	0	657	320	0.98	(0.71, 1.36)	−0.12	0.901	–	1.07	(0.75, 1.52)	0.37	0.708
Allocated treatment	0	657	320	0.83	(0.61, 1.12)	−1.21	0.225	–	0.91	(0.66, 1.26)	−0.57	0.566
Dose group	0	657	320	0.60	(0.17, 2.06)	−0.82	0.414	–	–	–	–	–
Beam modification	6	651	319	1.72	(1.21, 2.45)	3.03	0.002	–	1.85	(1.09, 3.14)	2.29	0.022
Treatment verification	2	655	319	1.49	(1.08, 2.05)	2.43	0.015	–	2.69	(1.55, 4.68)	3.51	<0.001
Phase I fields	2	655	319	1.22	(0.89, 1.67)	1.24	0.214	–	1.16	(0.82, 1.63)	0.84	0.403
Fractions imaged	0	657	320	1.87	(1.45, 2.40)	4.88	<0.001	–	1.56	(1.38, 1.77)	6.92	<0.001
Baseline bladder symptoms	6	651	319	0.80	(0.58, 1.10)	−1.40	0.161	–	0.78	(0.56, 1.10)	−1.4	0.161

T-stage and differentiation were not included in the multivariate models because they were jointly represented in the risk group. LMPA, low melting point alloy; MLC, multileaf collimator; EPI, electronic portal imaging.

∗ Category (reference category is depicted in bold): T-stage = **T1** vs T2 vs T3; Differentiation = **Good** vs moderate vs poor (based on differentiation or Gleason sum score, where available); Age = 46–62, 63–66, 67–70, 71 and over; Risk group = **Low** vs moderate (trial stratification factor); Allocated treatment = **74Gy/37 fractions** vs 64 Gy/32 fractions; Beam modification = **MLC** vs LMPA; Treatment verification = **EPI** vs film; Phase I fields = **three** vs four; Total dose = **<64Gy** vs 64 Gy+; Number of fractions reported = **0–3** vs 4–6 vs 7+.

**Table 6:** Patients with one or more displacement of 5 mm in weeks 3–6: univariate and multivariate logistic regression models

Category*	Data			Univariate models				Multivariate models				
	Miss	*n*	3 mm+ displacement	OR	(95% CI)	*z*	*P*	*n*	OR	(95% CI)	*z*	*P*
T-stage	2	639	365	1.13	(0.89, 1.44)	1.01	0.313	630	–	–	–	–
Differentiation	0	641	366	0.80	(0.58, 1.11)	−1.35	0.178	–	–	–	–	–
Age (quartile)	0	641	366	1.00	(0.87, 1.16)	0.06	0.953	–	0.94	(0.80, 1.11)	−0.68	0.493
Risk group	0	641	366	1.40	(1.00, 1.97)	1.94	0.052	–	1.21	(0.81, 1.80)	0.95	0.344
Allocated treatment	0	641	366	1.06	(0.78, 1.45)	0.39	0.698	–	1.06	(0.74, 1.52)	0.31	0.755
Dose group	0	641	366	0.75	(0.21, 2.61)	−0.46	0.649	–	–	–	–	–
Beam modification	5	636	363	2.99	(2.09, 4.29)	5.96	<0.001	–	1.10	(0.60, 2.00)	0.3	0.765
Treatment verification	1	640	366	2.94	(2.10, 4.10)	6.30	<0.001	–	3.12	(1.77, 5.48)	3.95	<0.001
Phase I fields	1	640	366	1.31	(0.95, 1.80)	1.65	0.099	–	0.77	(0.52, 1.13)	−1.33	0.185
Fractions imaged	0	641	366	3.83	(2.94, 5.00)	9.92	<0.001	–	1.70	(1.52, 1.91)	9.11	<0.001
Baseline toxicity	6	635	366	0.88	(0.64, 1.22)	−0.76	0.446	–	0.82	(0.56, 1.19)	−1.05	0.294

T-stage and differentiation were not included in the multivariate models because they were jointly represented in the risk group. LMPA, low melting point alloy; MLC, multileaf collimator; EPI, electronic portal imaging.

∗ Category (reference category is depicted in bold): T-stage = **T1** vs T2 vs T3; Differentiation = **Good** vs moderate vs poor (based on differentiation or Gleason sum score, where available); Age = 46–62, 63–66, 67–70, 71 and over; Risk group = **Low** vs moderate (trial stratification factor); Allocated treatment = **74Gy/37 fractions** vs 64 Gy/32 fractions; Beam modification = **MLC** vs LMPA; Treatment verification = **EPI** vs film; Phase I fields = **three** vs four; Total dose = **<64Gy** vs 64 Gy+; Number of fractions reported = **0–3** vs 4–5 vs 6–8 vs 9+.
